# Pain therapy to reduce perioperative complications

**DOI:** 10.1515/iss-2019-0008

**Published:** 2019-11-26

**Authors:** Stephan M. Freys, Esther Pogatzki-Zahn

**Affiliations:** Chirurgische Klinik, DIAKO Ev. Diakonie-Krankenhaus, Gröpelinger Heerstr. 406-408, 28239 Bremen, Germany; Department of Anesthesiology, Intensive Care and Pain Medicine, University Hospital Muenster, Münster, Germany

**Keywords:** complications, pain therapy, surgery

## Abstract

The incidence rates of adverse events secondary to any operation are a well-known problem in any surgical field. One outstanding example of such adverse events is postoperative pain. Thus, the incidence of acute postoperative pain following any surgical procedure and its treatment are central issues for every surgeon. In the times of Enhanced Recovery After Surgery (ERAS) programs, acute pain therapy became an increasingly well investigated and accepted aspect in almost all surgical subspecialties. However, if it comes to the reduction of postoperative complications, in the actual context of postoperative pain, surgeons tend to focus on the operative process rather than on the perioperative procedures. Undoubtedly, postoperative pain became an important factor with regard to the quality of surgical care: both, the extent and the quality of the surgical procedure and the extent and the quality of the analgesic technique are decisive issues for a successful pain management. There is growing evidence that supports the role of acute pain therapy in reducing postoperative morbidity, and it has been demonstrated that high pain scores postoperatively may contribute to a complicated postoperative course. This overview comprises the current knowledge on the role of acute pain therapy with regard to the occurrence of postoperative complications. Most of the knowledge is derived from studies that primarily focus on the type and quality of postoperative pain therapy in relation to specific surgical procedures and only secondary on complications. As far as existent, data that report on the recovery period after surgery, on the rehabilitation status, on perioperative morbidity, on the development of chronic pain after surgery, and on possible solutions of the latter problem with the institution of transitional pain services will be presented.

## Introduction

The incidence rates of adverse events secondary to any operation are a well-known problem in any surgical field. In the realm of general surgery, these incidences range from 3% to 53% [1], [2], [3]. One outstanding example of such adverse events is postoperative pain. Thus, the incidence of acute postoperative pain following any surgical procedure and its treatment are central issues for every surgeon [4]. In the times of Enhanced Recovery After Surgery (ERAS) programs, acute pain therapy became an increasingly well-investigated and accepted aspect in almost all surgical subspecialities [5], [6], [7]. However, if it comes to the reduction of postoperative complications, in the actual context of postoperative pain, “traditionally minded” surgeons tend to focus on their core competence, i.e. the operative process, rather than on perioperative procedures. Undoubtedly, postoperative pain became an important factor with regard to the quality of surgical care: both the extent and the quality of the surgical procedure, and the extent and the quality of the analgesic technique are decisive issues for a successful pain management [8]. At the same time, it should be underlined that the extent of the surgical trauma does not always parallel pain intensity and functional consequences and that some “small” surgeries (e.g. appendectomy, cholecystectomy, tonsillectomy) are also very painful [4].

It is therefore a key question: which is the hen, and which is the egg?

Is postoperative pain an indicator for a (developing) complication, which means that management of the complication will automatically lead to a simultaneous decline in pain severity, or is postoperative pain, itself, a complication on its own, which inversely means, that management of the postoperative pain will reduce the level of complications?

There is growing evidence that supports the role of acute pain therapy in reducing postoperative morbidity [9]. At the same time, a few reports are able to demonstrate that high pain scores postoperatively may contribute to a complicated postoperative course [10], [11], [12].

This overview shall comprise the current knowledge on the role of acute pain therapy with regard to the occurrence of postoperative complications. Most of the knowledge is derived from studies that primarily focus on type and quality of postoperative pain therapy in relation to specific surgical procedures and only secondary on complications. As far as existent, data that report on the recovery period after surgery, on the rehabilitation status, on perioperative morbidity, on the development of chronic pain after surgery, and on possible solutions of the latter problem with the institution of transitional pain services will be presented.

## Pain therapy and quality of recovery after surgery

The quality of postoperative recovery is commonly measured by either objective parameters such as length of recovery room or hospital stay or subjective parameters such as postoperative nausea and vomiting (PONV). In order to improve this aspect, an Australian group established a scoring system, referred to as the “Quality of Recovery-40 (QOR-40)” [13], to evaluate patients who underwent breast cancer surgery. When this score was applied, it was found to be higher in patients that received regional analgesia (i.e. a paravertebral block (PVB)) together with a reduced length of stay and a reduced PONV index [14]. A higher QOR-40 score was also found when dexmedetomidine or magnesium was administered systemically [15], [16].

Looking at the effect of regional analgesia in breast cancer surgery, it was also reported that the use of a single shot of PVB prior to surgery reduces recovery time from analgesia and the incidence of PONV [17]. A retrospective study pointed out that PVB could reduce both the length of the hospital stay and the incidence of PONV, provided the patient underwent mastectomy with immediate reconstruction [18]. Another retrospective study showed that PVB reduced opioid consumption as well as the length of the hospital stay [19].

Another technique for regional analgesia during breast surgery was implemented with the pectoral nerves (PECS) types 1 and 2 blocks. Their application, together with general analgesia, leads to improved analgesia and a decreased incidence of PONV and a shorter hospital stay [20].

Looking at the effect of systemic analgesia in breast cancer surgery, a literature review reports on intravenous injection of clonidine [21], which resulted in a reduced analgesic consumption and PONV incidence, while intravenous dexmedetomidine [15] reduced both the postsurgical tramadol consumption and PONV incidence and increased the QoR-40 score after surgery.

In summary the data available in breast cancer surgery demonstrate that regional analgesia, including PVB and local anesthetic infiltration, is associated with higher QoR-40 score and reduced PONV incidence and earlier discharge compared to an opioid-based analgesia [22].

## Pain therapy and postoperative rehabilitation

It is assumed that an effective pain treatment will facilitate and promote early postoperative rehabilitation with all benefits from better physiotherapy to early discharge and return to work. Unfortunately, only few data are available [9].

A randomized controlled trial reporting on knee surgery found that epidural analgesia (EA) and peripheral nerve blocks (PNB) were a combination of methods that, compared with intravenous patient controlled analgesia (PCA), improved knee flexion and reduced the length of rehabilitation [23]. The same combination, EA and continuous PNB, were investigated in patients undergoing unilateral total knee arthroplasty [24]. When compared with intravenous morphine PCA, a significantly better knee flexion at 6 weeks, a faster ambulation, and a shorter hospital stay were reported. Interestingly this short-term effect could not be reported later on, and the outcome at 3 months was not affected.

One randomized, double-blind, placebo-controlled trial on patients undergoing hip fracture surgery was not able to demonstrate improvement in recovery of physical independence for the EA patients [25]. One year before, another group similarly found no advantage for PNB with regard to an early rehabilitation [26]. Another report on interscalene block in a cohort of randomized patients undergoing open shoulder surgery was able to show that interscalene block appeared to be superior to intravenous PCA with regard to pain during physiotherapeutic exercise, while function during the early rehabilitation was not improved [27].

Besides a systemic and/or catheter-bound regional acute pain therapy, the concept of a local pain therapy was established throughout the past years. Such a localized pain therapy may be performed in a single-shot technique with long-lasting local anesthetics in small laparoscopy incisions either immediately pre- or postoperatively. A good alternative, however, at present that is less known, is a continuous postoperative wound infiltration via small catheters that will be inserted into the wound at the end of the operation. Long-acting local anesthetics may be administered with a primary bolus of 5–10 mL with a subsequent continuous infusion flow rate of 5–10 mL/h by way of self-emptying elastomeric pump systems. Ideal incisions for this technique are medium-sized incisions in conventional hernia surgery, Pfannenstiel incisions, or incisions for breast surgery.

A recent meta-analysis reports on a comparison of postoperative epidural analgesia with a catheter-bound continuous wound infiltration [28]. The available literature with regard to continuous wound infiltration was scanned according to the Cochrane method, and 16 randomized trials were extracted. Despite the fact that a significant heterogeneity with often poor data as to dosage and kind of medication as well as small case numbers, the following three conclusions were drawn:

– A continuous wound infiltration leads to a significantly lower ratio of postoperative hypotension.
– Complications such as abscess formation, bleeding, ileus, nausea and vomiting, urinary tract infection, wound infection, and itching were equal in both analgesic techniques.
– In the epidural analgesia group, the postoperative pain score was significantly lower at rest and at mobilization compared to the group of patients with continuous wound infiltration.

Another meta-analysis focused on the position of the postoperatively administered wound catheter: preperitoneal vs. subcutaneous [29]. Twenty-nine randomized trials were identified; however, only one trial reported on a direct comparison in 60 patients between preperitoneal and subcutaneous catheter positions. The remaining 28 trials compared the different catheter positions with either epidural analgesia or placebo controls.

Two clear statements were reported:

– There is an indirect advantage for the preperitoneal catheter position, as the reported postoperative pain control was similar to the group of patients with epidural analgesia.
– Postoperative mobilization, patient satisfaction, hypotension as side effect, and analgetic consumption was less in the group of patients with a preperitoneal catheter position.

In essence, the available data are only able to give a hint toward an improvement in the early postoperative rehabilitation, thus, leading to an avoidance of typical complications in this time period. No conclusive or long-term data exist.

## Pain therapy and perioperative morbidity

Without specific data on each surgical subspecialty, perioperative morbidity is the single most prominent problem in possible existing postoperative adverse events. Perioperative morbidity comprises problems with respiratory, coagulatory, intestinal, and hormonal stress-induced function disorders.

A recently published study on the association between postoperative pain and 30-day postoperative complications highlights the problem [12]. Consecutive patients (1.014) undergoing scheduled surgery in a 2.5-year period in a Dutch University Hospital were assessed as to the intensity of pain (Movement Evoked Pain score on the Numerical Rating Scale (NRS-MEP)) and the patient’s opinion whether the pain was acceptable or not. The outcome was the presence of a complication using the Clavien-Dindo Classification of Surgical Complications [30]. The results were: 55% of the patients experienced moderate-to-severe pain on the first postoperative day; the overall complication rate was 34%. The proportion of patients with postoperative complications increased from 25% for NRS-MEP=0 to 45% for NRS-MEP=10. Patients who classified their pain as unacceptable had statistical significant more complications (adjusted odds ratio=2.17). Besides these data, the authors were able to show that complications that could be linked to pain through a plausible mechanism showed a stronger positive association with pain scores than other complications. In addition, hospital-acquired infections were strongly associated with higher pain levels during the early postoperative phase.

The results of this study reflect a common knowledge: postoperative pain impairs both physical mobilization and pulmonary mobilization, i.e. coughing, thus leading to a higher risk of respiratory complications. A delayed removal of urinary catheters secondary to pain-related delayed mobilization may increase the incidence of urinary tract infections. Finally, a delayed intestinal function may be secondary to postoperative pain and/or adverse effects of analgesia. The authors conclude that their findings support the hypothesis of a causal relationship between postoperative pain and complications after surgery, leading to the advice that personalized analgesia in modern perioperative care is a central issue.

Within the past two decades, it is has become evident that EA is able to reduce a good amount of the above-mentioned postoperative problems. The effects of different anesthetic and postoperative analgesic techniques on perioperative complications were evaluated in a systematic review [31]. A clear-cut statement pointed out that the majority of the evidence favored EA compared with general anesthesia alone in high-risk patients or patients undergoing major vascular surgery. At the same time, there was a consistent recommendation that the use of EA promoted the resolution of postoperative ileus after major abdominal surgery. However, for other forms of regional analgesia, intravenous PCA, and multimodal systemic analgesia, the review failed to find evidence for a clinically important reduction in the incidence of postoperative complications.

Another meta-analysis focused on the protective effects of epidural analgesia on pulmonary complications after abdominal and thoracic surgery [32]. Covering the 25-year period from 1971 to 1996, it could be demonstrated that EA has a protective effect on the incidence of postoperative pneumonia. While there was a general decrease in postoperative pneumonia in the time span looked upon (the incidence dropped from 34% to 12% with systemic analgesia), the incidence in patients with EA amounted to 8% only. Additional important findings were that EA lowered the need for prolonged postoperative ventilation or reintubation, improved lung function, and blood oxygenation. At the same time, this review pointed out the well-known negative side effects of EA with an increased risk of hypotension, urinary retention, and pruritus.

The same group published a systematic review and meta-analysis of randomized controlled trials 6 years later in order to elicit the impact of epidural analgesia on mortality and morbidity after surgery [33]. In 10 out of 125 trials evaluated, mortality was reported as the primary or secondary endpoint. In patients who received EA in addition to general anesthesia, the risk of death decreased significantly from 4.9% to 3.1%. EA significantly decreased the risk of atrial fibrillation, supraventricular tachycardia, deep vein thrombosis, respiratory depression, atelectasis, pneumonia, ileus, and PONV, and also improved a recovery of bowel function. Again, the side effects of EA were reported with a significantly increased risk of arterial hypotension, pruritus, urinary retention, and motor blockade.

Again, looking upon the effect of a combination of general anesthesia and EA, a retrospective cohort study on patients with intermediate and high-risk noncardiac surgery found that EA was associated with a statistical significant reduction in the 30-day-mortality [34]. However, the number needed to treat was quite high with 477 patients having to undergo noncardiac surgery in order to prevent one perioperative death from general anesthesia alone.

A quite recent study investigated patients undergoing any type of colectomy with or without EA using the American College of Surgeons National Surgical Quality Improvement Program (NSQIP) to assess any association between EA (versus non-EA) and complications after colectomy. Patients, 4.176, with EA were matched 1:4 via propensity score to 16.704 non-EA patients undergoing colectomy [35]. The primary outcome was the incidence of cardiopulmonary complications; the secondary outcomes included neurologic, renal, and surgical complications as well as length of hospitalization. There was no significant association between EA and both primary and secondary outcomes. However, an interesting finding was the fact that in the subgroup of open (conventional) colectomies, EA was associated with fewer cardiopulmonary complications and a shorter length of hospitalization.

Putting together the available information on the role of EA, there is undoubtedly a favorable effect on the postoperative outcome, even if this effect may be restricted to major surgery performed on intermediate or high-risk patients.

The possible advantages of regional analgesic techniques have to be balanced to the inherent risk of such techniques if looking upon postoperative complications. There are two excellent surveys which allow for a reasonable opinion: A National Audit Project of the Royal College of Anaesthetists entailed a 2-week national census, which identified 707.455 central neural blockades performed over 1 year in the UK National Health Service [36]. All major complications that occurred during this period (vertebral canal abscess or hematoma, meningitis, nerve injury, spinal cord ischemia, fatal cardiovascular collapse, and wrong route errors) were reported. The incidence of permanent injury due to central neural blockade was “pessimistically’” 0.0042% and “optimistically” 0.002%. The incidence of paraplegia or death was “pessimistically” 0.0018% and “optimistically” 0.0007%. Two-thirds of the initially disabling injuries resolved fully.

Quite similar numbers were derived from a prospective analysis of 18.925 postoperative patients receiving patient-controlled EA, intravenous PCA, continuous brachial plexus block, and continuous femoral/sciatic nerve block in a German University Hospital [37]. Epidural hematoma occurred in 1 of 4.741 patients (0.02%), without permanent neurological sequelae. Epidural abscess was observed in 2:14.233 patients (0.014%), one with a permanent neurological deficit and another with meningitis with complete resolution. Transient severe neurological deficit occurred in 2:3.111 patients (0.006%) with PNB, with no cases of permanent damage.

In summary, neurological damage and even more permanent damage is extremely rare after regional analgesic techniques. These rather rare complications should not counterweigh the advantages of such techniques with regard to the option of reducing postoperative complications.

## Pain therapy and chronic pain after surgery

More than 10 years of research undoubtedly demonstrate high incidences of persistent, chronic pain (CP) that resides after many surgical procedures [38]. One large European prospective multicenter trial indicated a mean incidence of 11.8% patients with moderate-to-severe CP at 12 months after surgery [39]. Although the incidence of severe CP (with 6 or higher, NRS from 0 to 10) was rather low (2.2% patients), this means that 2 out of 100 patients after any surgical procedure has a major pain problem 1 year after surgery that reduces the quality of live dramatically [39]. Other studies are in line with this and indicate that severe chronic postsurgical pain that negatively affects the patient’s quality of life is in the range of 2%–15% [38], [39], [40]. Thus, every patient with pain that lasts years after surgery (maybe lifelong) needs to be considered as it is one too many. This was recognized in recent years, and CP after surgery is now scheduled to be included in the upcoming version of the International Classification of Diseases, 11th Revision (ICD-11), which results from the joint efforts of the World Health Organization (WHO) and the International Association for the Study of Pain (IASP) [41]. This will increase recognition by health care providers as well as researchers and hopefully reduces the burden of patients with CP after surgery in the future.

A prerequisite for the detection of patients at risk for the development of CP after surgery is a strict pain assessment and assessment of pain-related functional interferences in the early postoperative phase. If pain (and its consequences) is not assessed, and/or – perhaps even more important – if assessment is not embedded in a setting of standardized clinical pathways, which triggers well-defined analgesic techniques, deficits of pain management will remain undetected.

From many studies, it is clear that not every surgical procedure has the same risk of CP month to years thereafter [42]. Although studies investigating different surgical procedures with the same methodology are rare, there are clear indications for a higher incidence of CP in some surgeries (e.g. thoracic surgery, breast surgery, amputation) and lower incidence in others(large joint surgeries, abdominal surgical procedures, surgery of the pelvic organs) [42]. Interesting is the fact that the group of surgical procedures with higher incidences may have a higher percentage of patients that suffer from neuropathic pain. In the European multicenter trial, neuropathic pain was more frequent in patients reporting higher CP than those patients reporting more mild CP [42]; other studies are confirming this finding [40]. Neuropathic pain *per se* is a devastating symptom, and treatment still remains difficult and often unsuccessful [43]. Thus, prevention of the development of (neuropathic) pain after surgery is an essential goal but difficult to reach.

The risk factors associated with the development of CP after surgery are diverse; however, most of them are associated with the patients’ preoperative status (young age, psychosocial factors like stress and capacity overload, sleep disturbance, and preoperative pain) as well as perioperative pain-related symptoms and pain management [44], [45]. The latter aspect is of major importance. For example, patients with an increased slope of recovery from pain (these are the patients with increased pain ratings in the first postoperative days instead of a normal decrease in pain) had a significant higher rate of pain 3 months after total knee surgery [46]. Also, early acute neuropathic pain-like symptoms (characteristics like burning, painful cold, electric shock, tingling, or numbness surrounding the wound) seem to increase the risk of CP with a neuropathic component [47]. In addition, CP after cholecystectomy was related to pain experiences during the first week after surgery [48]. Finally, areas of sensitive skin surrounding the abdominal wound (area of hyperalgesia) was larger in patients that developed CP compared to those without CP after surgery [49], [50]. Reducing the hypersensitivity during and shortly after surgery was able to reduce the incidence of chronic pain [49], [50], [51].

The latter findings exemplify three important aspects:

1. There is a chance to identify patients with a high risk of developing CP after surgery, and a number of risk scores are currently under development [52], [53]. These patients might be treated more precisely early with preventative treatment options able to reduce CP after surgery. However, there are not many preventative options available to date. One might be the reduction of acute pain *per se* with more efficient analgesic approaches. Here, regional anesthesia techniques have been more favorable than drugs (e.g. opioids) [54]. A systematic Cochrane review focused on the effect of a perioperative intravenous lidocaine infusion compared to either placebo to no treatment or to epidural analgesia with regard to postoperative pain und postoperative recovery in patients undergoing different surgical procedures [54]. Three conclusions were drawn:
  – It remains unclear whether a perioperative intravenous lidocaine administration offers an advantage compared to placebo or no treatment with regard to postoperative pain score in the early postoperative phase, to gastrointestinal recovery, to postoperative nausea, and to opioid consumption.
  – The quality of evidence was limited by the inconsistency, the inaccuracy, and the quality of currently available trials.
  – There is no sufficient evidence as to the optimal intravenous lidocaine dosage and duration of administration in comparison to epidural analgesia.
2. Specific preventative treatment options might be given perioperatively; however, here, only ketamine seems to be effective in preventing (some aspects of) CP after surgery [55].
3. There is a very interesting and new approach to prevent CP after surgery by a multidisciplinary team in the hospital as well as after discharge (see below); this service has been introduced punctually in some countries in single hospitals, and the first results are promising [56], [57].

## Transitional pain service

Many studies suggest that chronic pain after surgery is multifactorial (see above). Thus, prevention and treatment might only work using a multidisciplinary approach. Furthermore, prevention and treatment might need to continue for a while after the patient has left the hospital. In order to decrease CP after surgery effectively and sustainably, a comprehensive, multi-disciplinary and inter-sectoral working facility dedicated to prevent and treat CP after surgery was proposed [56], [57] and is now starting to be incorporated in clinical practice. The Acute Pain Service Out Patient Clinic (APS-OPC) in Helsinki and the Toronto General Hospital Transitional Pain Service are two examples developed in 2012 and 2014, respectively [58], [59], [60]. In both centers, a multidisciplinary team providing care pre-operatively, post-operatively, and after discharge when patients have returned home was established. The three main goals of the services are to:

1. identify patients with an increased risk for developing chronic post-surgical pain,
2. provide adequate pain medication for patients (including rather a reduction of opioids than a continuation), and
3. offer psychological and/or physiotherapeutic treatment to provide support where negative aspects limit recovery and functioning [58], [59], [60].

The first results indicate an impressive reduction in opioid use after surgery by such a multidisciplinary approach [58]. Such an effect is of major importance in face of the huge opioid epidemic in the US, which was partly a result of postoperative long-term overuse of opioids [61]. Because of this over-prescribing attitude, an expert panel involving six relevant stakeholder groups (surgeons, pain specialists, outpatient surgical nurse practitioners, surgical residents, patients, and pharmacists) used a three-step modified Delphi method to develop consensus ranges for outpatient opioid prescription. As a result, three primary recommendations were consented [62]:

1. to provide patients with instructions to maximize the use of nonopioid analgesics;
2. the minimum number of opioid tablets to prescribe after each procedure is 0, depending on procedure and patient characteristics;
3. the maximum number of opioid tablets to prescribe varies by procedure, but should not exceed 20 tablets.

Together, for in and outpatient surgeries, appropriate use of analgesics together with, e.g. after inpatient major procedures with a high risk of prolonged and chronic pain after surgery, a multidisciplinary preventative approach, ideally by having a “transitional pain service” in place, would be of great advantage. However, future effort needs to be taken to show the medical as well as socioeconomic benefits of such a service to appeal hospitals and politics to invest in this.

## Supplementary Material

The article (https://doi.org/10.1515/iss-2019-0008) offers reviewer assessments as supplementary material.
