# Supplementary material for: Pain therapy to reduce perioperative complications
Source: Innov Surg Sci. 2019 Nov 26;4(4):158–66. doi: 10.1515/iss-2019-0008 (PMC8059349; doi:10.1515/iss-2019-0008)
Supplement: Supplementary file 1 [file iss-04-20190008-s001.pdf]

Reviewer Assessment

Stephan M. Freys* and Esther Pogatzki-Zahn

Pain therapy to reduce perioperative complications

https://doi.org/10.1515/iss-2019-0008
Received April 22, 2019; accepted September 16, 2019

***Corresponding author: Prof. Dr. Stephan M. Freys,** Chirurgische Klinik, DIAKO Ev. Diakonie-Krankenhaus, Gröpelinger Heerstr. 406-408, 28239 Bremen, Germany, E-mail: s.freys@diako-bremen.de, www.diako-bremen.de

Reviewers' Comments to Original Submission

Reviewer 1: Prof. Winfried Meißner

Jun 11, 2019

**Reviewer Recommendation Term:**	**Revise with Major Modifications**
**Overall Reviewer Manuscript Rating:**	**40**
Is the subject area appropriate for you?	4
Does the title clearly reflect the paper's content?	4
Does the abstract clearly reflect the paper's content?	4
Do the keywords clearly reflect the paper's content?	3
Does the introduction present the problem clearly?	4
Are the results/conclusions justified?	4
How comprehensive and up-to-date is the subject matter presented?	4
How adequate is the data presentation?	- N/A
Are units and terminology used correctly?	- N/A
Is the number of cases adequate?	- N/A
Are the experimental methods/clinical studies adequate?	- N/A
Is the length appropriate in relation to the content?	4
Does the reader get new insights from the article?	4
Please rate the practical significance.	5 - High/Yes
Please rate the accuracy of methods.	- N/A
Please rate the statistical evaluation and quality control.	- N/A
Please rate the appropriateness of the figures and tables.	- N/A
Please rate the appropriateness of the references.	4
Please evaluate the writing style and use of language.	4
Please judge the overall scientific quality of the manuscript.	3
Are you willing to review the revision of this manuscript?	Yes

Open Access. © 2019 Freys S.M., Pogatzki-Zahn E., published by De Gruyter. [CC BY] This work is licensed under the Creative Commons Attribution 4.0 Public License.

Comments to Author:

The authors present a comprehensive overview over the impact of perioperative pain and pain management on surgical outcomes. The article summarizes current knowledge on this topic from the surgical perspectives. It specifically addresses the different views on pain: Pain as a symptom and the consequences of surgical procedures on one side, and pain as a problem of its own and a potential reason of complications on the other side. The article highlights that the first "view" often leads to underestimation of pain and under-treatment of patients, neglecting the fact that pain can not always be controlled by surgical processes, and may worsen surgical outcomes.

The paper has not used a systematic literature research approach but has the character of narrative review. This might explain why certain surgeries (e.g., breast cancer surgery, joint surgery) are over-represented. Perhaps the authors could mention that the extent of the surgical trauma does not always parallel pain intensity and functional consequences, and that some "small" surgeries (e.g., appendectomy) are also very painful.

The article covers the most important issues of perioperative pain management, including regional analgesia and some forms of systemic pain management. However, the role of local (wound) infiltration (and peritoneal instillation) of local anesthetics is hardly mentioned. I suggest to add this simple but - at least for immediate postoperative period - rather effective intervention because it is something which has to be done by the surgeon and can not made up leeway.

I recommend to mention intravenous lidocaine as a potential prevention of chronic postsurgical pain, as the latest Cochrane review highlights its potential role after some surgeries (e.g., breast cancer surgery and thoracotomies) [1].

Another important issue of perioperative pain management is the assessment of pain and its risk factors. This topic is discussed in the context of chronic pain after surgery. I recommend adding a short paragraph underlining the importance of pain assessment and of pain-related functional interference also in the early phase after surgery. If pain (and its consequences) is not assessed, and/or - perhaps even more important - if assessment is not embedded in clinical pathways which trigger pain management, deficits of pain management will remain undetected.

Specific comments:
Page 4:
„With regards to regional analgesia there are also few data available:": In the sentence before, RA was addressed as well.
Page 8:
„Here, regional anesthesia techniques have been more favorable than drugs": add iv lidocaine (see comment above).

1. Weinstein, E.J., et al., Local anaesthetics and regional anaesthesia versus conventional analgesia for preventing persistent postoperative pain in adults and children. Cochrane Database Syst Rev, 2018. 4: p. CD007105.

Reviewer 2: Wolfgang Hiller

Jul 25, 2019

**Reviewer Recommendation Term:**	**Accept**
**Overall Reviewer Manuscript Rating:**	80
Is the subject area appropriate for you?	5 - High/Yes
Does the title clearly reflect the paper's content?	5 - High/Yes
Does the abstract clearly reflect the paper's content?	5 - High/Yes
Do the keywords clearly reflect the paper's content?	5 - High/Yes
Does the introduction present the problem clearly?	5 - High/Yes
Are the results/conclusions justified?	4
How comprehensive and up-to-date is the subject matter presented?	5 - High/Yes
How adequate is the data presentation?	4
Are units and terminology used correctly?	5 - High/Yes
Is the number of cases adequate?	- N/A
Are the experimental methods/clinical studies adequate?	- N/A
Is the length appropriate in relation to the content?	5 - High/Yes
Does the reader get new insights from the article?	3

Please rate the practical significance.	5 - High/Yes
Please rate the accuracy of methods.	5 - High/Yes
Please rate the statistical evaluation and quality control.	- N/A
Please rate the appropriateness of the figures and tables.	- N/A
Please rate the appropriateness of the references.	5 - High/Yes
Please evaluate the writing style and use of language.	5 - High/Yes
Please judge the overall scientific quality of the manuscript.	4
Are you willing to review the revision of this manuscript?	Yes

Comments to Author:
The paper gives a comprehensive overview on the topic, which is of major importance for every surgeon. The language is flawless and nicely written. I have no suggestions concerning changes which have to be made and therefore strongly recommend publication.

Authors' Response to Reviewer Comments

Sep 3, 2019
We have revised the manuscript and have included all proposed changes made by reviewer # 1:

1. it has been added “that the extent of the surgical trauma ....”
2. “the role of local (wound) Infiltration” has been added
3. a short paragraph “underlining the importance of pain assessment...” has been added
4. the role of “intravenous lidocaine” has been added
5. the mentioned Cochrane Review was already part of the literature, now: (54)

Editor Comments to Final Decision

The manuscript has improved a lot by minor revisions made. It should be accepted for publication without further review.
